# Supplementary material for: Insights on Proteomics-Driven Body Fluid-Based Biomarkers of Cervical Cancer
Source: Proteomes. 2022 Apr 29;10(2):13. doi: 10.3390/proteomes10020013 (PMC9149910; doi:10.3390/proteomes10020013)
Supplement: Supplementary file 1 [file proteomes-10-00013-s001.zip › Supplementary Table S1_ List of proteins used for metadata analysis.pdf]

Supplementary Table S1: List of proteins used for metadata analysis

Literature referred	Van Raemdonck et al.	Starodubtseva et al.	Van Ostade et al.
Criteria of selection	Increased levels in CVF from women with adenocarcinoma: Increased levels in CVF from women with precancerous lesions with stringent statistical selection based on Pearson Chi squared and Mann-Whitney U tests ($p < 0.05$) : Exclusive occurrence in CVF from women with precancerous lesions and described to be involved in cervical cancer	Explicit and implied upregulation in (CANCER, HSIL, and LSIL) vs NILM	Proteins with a statistically significant difference in frequency between the healthy and precancerous group and those with a statistically significant difference in abundance between the healthy and precancerous group (Table 1 and Table 2 of Van Ostade et al., 2017). Those proteins with higher parameter values in both tables are enlisted.
Uniprot IDs	O43707	P02763	Q13984
	P00558	P02765	Q969X2
	P29373	P01023	P62258
	P29508	P01024	P61158
	P43490	P02675	O43707
	P61158	P02679	P07355
	P62258	P05155	P06576
	Q5VTE0	P02774	P29373
	Q9UIV8	P68133	P43490

	P06576	P62736	P00558
	P07355	P68032	Q5VTE0
	P14618	P63267	P14618
	P00338	P07355	P29508
		D1MGQ2	P01019
		P04004	P27348
		P02671	P09525
		O43707	P07858
		Q9HC84	P13987
		Q92876	P00450
		O43240	P06396
			Q9HC84
			P60660
			P18669
			P30101
			P25815

			Q9UIV8
			P40121
			P14174
			Q14116
			P26583
			P04179
